# Dietary Iodine Intake and Sources among Residents in Zhejiang Province 10 Years after Reducing Iodine Concentration in Iodized Salt

**DOI:** 10.3390/nu16132153

**Published:** 2024-07-05

**Authors:** Jiaxin He, Lichun Huang, Chenyang Liu, Zhe Mo, Danting Su, Simeng Gu, Fanjia Guo, Yuanyang Wang, Zhijian Chen, Xiaofeng Wang, Ronghua Zhang, Xiaoming Lou, Guangming Mao

**Affiliations:** 1 Department of Environmental Health, Zhejiang Provincial Center for Disease Control and Prevention, 3399 Binsheng Road, Hangzhou 310051, China; jxhedyx@163.com (J.H.); zhmo@cdc.zj.cn (Z.M.); smgu@cdc.zj.cn (S.G.); fjguo@cdc.zj.cn (F.G.); yywang@cdc.zj.cn (Y.W.); zhjchen@cdc.zj.cn (Z.C.); xfwang@cdc.zj.cn (X.W.); xmlou@cdc.zj.cn (X.L.); 2 Department of Nutrition and Food Safety, Zhejiang Provincial Center for Disease Control and Prevention, 3399 Binsheng Road, Hangzhou 310051, China; wlch12688@163.com (L.H.); sdting12368@sina.com (D.S.); zhrh128@163.com (R.Z.); 3 School of Public Health, Zhejiang University, Hangzhou 310058, China; 22118893@zju.edu.cn

**Keywords:** dietary iodine, contribution rate, iodized salt, seafood

## Abstract

We aimed to assess dietary iodine intake and sources in Zhejiang Province a decade after a reduction in iodine concentration in iodized salt. Three-day 24 h dietary recall and household weighing were used, complemented by “Chinese Food Composition” data. Household water and salt samples were collected from 5890 residents and analyzed. Differences in iodized salt consumption rates were observed across the following regions: inland (84.20%), subcoastal (67.80%), and coastal (37.00%) areas. The median (P_25_, P_75_) iodine concentration in water and diet were 2.2 (0.9, 4.0) μg/L and 142.05 (58.94, 237.11) μg/d, respectively, with significant regional differences in dietary concentration (inland [185.61 μg/d], subcoastal [153.42 μg/d], and coastal [75.66 μg/d]). Males (149.99 μg/d) and iodized salt consumers (191.98 μg/d) had a significantly higher dietary iodine intake than their counterparts. Regions were ranked as follows based on the proportions of individuals meeting the recommended dietary iodine intake: inland (69.40%), subcoastal (56.50%), and coastal (34.10%) areas. Dietary sources included salt (48.54%), other foods (32.06%), drinking water (8.84%), laver (4.82%), kelp (3.02%), and other seafood (2.32%). The qualified iodized salt consumption rate was significantly lower than the national standard. Zhejiang Province should continue implementing measures to control iodine deficiency through salt iodization, education efforts, and increasing the qualified iodized salt consumption rate.

## 1. Introduction

Iodine is an essential trace element, and its intake is intricately linked to human health. As an essential micronutrient required for synthesizing thyroid hormones, iodine plays a pivotal role in regulating metabolism and facilitating growth and development in the body [1,2]. Both the deficiency and excessive levels of iodine can precipitate various diseases [3]. Prolonged excessive intake disrupts self-regulatory mechanisms and leads to functional imbalances, resulting in a range of conditions, such as hyperthyroidism and autoimmune thyroiditis [4]. Iodine deficiency can lead to disorders, such as stillbirths, congenital abnormalities, cretinism, and increased infant mortality, as well as impaired cognitive function and stunted growth in children and adolescents. In adults, iodine deficiency may induce hyperthyroidism, while pregnant women face a heightened risk of spontaneous abortion [5]. In China, combating iodine deficiency disorders (IDDs) has long been a pressing public health challenge.

Since China initiated the Universal Salt Iodization (USI) program in 1995, significant strides have been made toward improving iodine nutrition nationwide [6,7], with Zhejiang Province also achieving notable success in preventing and controlling IDDs [8]. Nevertheless, with its economic development in recent years, there have been substantial shifts in the dietary patterns and habits of urban and rural residents in Zhejiang Province, posing new challenges to IDD prevention and control efforts. On one hand, with the effective implementation of salt iodization, severe iodine deficiency-related conditions, such as cretinism, have become rare. However, thyroid-related diseases associated with mild iodine deficiency have become less recognized [9]. Consequently, residents’ awareness of the hazards of iodine deficiency is gradually diminishing, leading to a waning sense of urgency in implementing prevention and control efforts. This has led to some residents discontinuing the use of iodized salt, thereby heightening the risk of IDDs. On the other hand, in line with the “National Food Safety Standards—Iodine Content of Salt” (GB 26878-2011) [10], the iodine concentration in salt has gradually decreased since 2012 in China. Correspondingly, the concentration in Zhejiang Province dropped from 35 ± 30 mg/kg to 18–33 mg/kg over a 10-year period until 2022. Additionally, the introduction of the “Reform Plan for the Salt Industry System” [11] has expanded the variety of salt products available in the market, leading to the infiltration of non-iodized salt. Consequently, there has been a noticeable decline in the consumption rate of qualified iodized salt among residents in Zhejiang Province in recent years, prompting changes in the population’s iodine nutrition status.

Approximately 80% of iodine intake in the human body is derived from food, 10–20% from drinking water, and <5% from the air [12]. Dietary iodine intake serves as a pivotal measure for assessing both the individual- and population-level iodine nutrition status, as well as for investigating the prevalence of thyroid diseases [13]. It is considered the most direct biological marker of iodine nutrition status in both populations and individuals [14,15]. The adequacy of iodine intake hinges on the consumption of iodine-rich foods and their iodine content. A deficiency of iodine in the diet directly results in iodine deficiency in the body [16].

Therefore, to further understand the dietary iodine status among residents in Zhejiang Province, a comprehensive survey on province-wide dietary iodine intake was conducted in 2022. This endeavor was aimed at evaluating the dietary iodine status among residents and to compare the contributions of iodized salt, drinking water, laver, kelp, and other foods to dietary iodine intake. The initiative sought to provide the public with a thorough and accurate understanding of the current state of iodine supplementation and the significance of various foods. It also endeavored to create a foundation for the effective implementation of a strategy involving “tailored measures, targeted guidance, and scientific iodine supplementation”, ensuring the eradication of IDDs while preventing the harms related to excessive iodine intake, thereby safeguarding the health of residents. In this study, we aimed to assess dietary iodine intake and sources among the residents of Zhejiang Province a decade after a reduction in iodine concentration in iodized salt using data from the 2022 survey.

## 2. Materials and Methods

### 2.1. Study Population

This cross-sectional analysis was based on data from the 2022 nutritional survey conducted in Zhejiang Province. By employing a stratified, multi-stage, systematic, random sampling approach, a representative sample reflecting Zhejiang Province’s demographic composition was established, spanning 16 surveyed counties (districts, cities). First, the province was categorized into three groups based on distinct geographical locations: coastal, subcoastal, and inland areas. Subsequently, leveraging the population composition ratio of selected monitoring points, six committees of residents (villagers) were chosen from each monitoring point, with 30 households selected from each committee through systematic sampling for dietary assessments.

### 2.2. Estimation of Dietary Iodine Intake

The questionnaire was primarily used to collect information on sociodemographic factors (age, ethnicity, marital status, education, occupation, etc.), dietary patterns, health status, and lifestyle choices. Trained surveyors, who underwent standardized technical training, conducted a 24 h dietary recall survey over 3 consecutive days; all permanent members of the surveyed households participated. During the survey, all foods consumed at home (or in school cafeterias) and in outside establishments were documented over 3 consecutive days, 24 h a day. Additionally, a household food weighing method was employed to assess the consumption of various cooking oils and condiments (such as monosodium glutamate, table salt, and soy sauce) over 3 consecutive days. By combining these data with the “Chinese Food Composition” data [17], the iodine content in each food item was determined in order to calculate individual dietary iodine intake. Meanwhile, samples of household drinking water and edible salt were collected, and their iodine contents were measured using cerium sulfate catalytic spectrophotometry and the direct titration method, respectively.

As shown in Table 1, references for the estimated average requirement (EAR), recommended nutrient intake (RNI), and tolerable upper intake level (UL) for iodine were obtained from the “Dietary Reference Intakes for Chinese Residents” [18] based on different age groups and physiological conditions. 

Household edible salt was categorized into three groups according to the “National Food Safety Standards—Iodine Content of Salt” [10]: non-iodized salt (iodine content < 5 mg/kg), non-compliant iodized salt (iodine content < 18 mg/kg or > 33 mg/kg), and qualified iodized salt (iodine content 18–33 mg/kg). The coverage rate of iodized salt was calculated as the proportion of household salt samples with iodine content ≥ 5 mg/kg out of the total number of tested samples. Iodine-deficient areas (median water iodine < 40 μg/L) and iodine-adequate areas (40–100 μg/L) (based on administrative villages) were classified according to the “Definition and Demarcation of Iodine Deficient Areas and Iodine Adequate Areas” [19].

### 2.3. Statistical Analysis

Data analysis was conducted using SPSS 21.0 statistical software (IBM Corporation, Armonk, NY, USA). Normality was assessed using the Kolmogorov–Smirnov (KS) test. Dietary iodine intake and salt iodine content were expressed as the median and quartile range (P_25_, P_75_). Regional differences were examined using the Kruskal–Wallis H test for multiple independent samples, while demographic comparisons, such as those between urban and rural areas and between sexes, were performed using the Mann–Whitney U test for two independent samples. Pairwise comparisons were executed using the Nemenyi test. Count data were presented as rates/composition ratios, with inter-group differences evaluated via the χ^2^ test. A trend analysis was performed using the χ^2^ trend test. All tests were two-tailed, and the significance level was set at *p* < 0.05.

## 3. Results

### 3.1. Iodine Contents in Water and Salt in Zhejiang Province

As shown in Table 2, all 16 surveyed points relied on centralized water supply systems. A total of 251 samples of drinking water were collected, with a median (P_25_, P_75_) water iodine content of 2.2 (0.9, 4.0) μg/L.

Furthermore, 3047 samples of household salt were collected, comprising 966 samples of non-iodized salt, 125 samples of non-compliant iodized salt, and 1956 samples of qualified iodized salt. The coverage rate of iodized salt was 68.30%, and the consumption rate of qualified iodized salt was 64.20%. The median (P_25_, P_75_) iodine content in the household salt of residents in Zhejiang Province was 22.08 (0.00, 24.51) mg/kg, with iodized salt having a median iodine content of 23.70 mg/kg.

Significant regional variations were observed in household iodized salt content (all *p* < 0.001), with the trend analysis demonstrating statistical significance. The proportions of households using qualified iodized salt by region, from the largest to the smallest, were as follows: inland (84.20%), subcoastal (67.80%), and coastal (37.00%) areas (*χ^2^* _trend_ = 504.765, *p* _for trend_ < 0.001). Conversely, the proportions of households using non-iodized salt by region were as follows: coastal (58.40%), subcoastal (28.40%), and inland (11.90%) areas (*χ^2^* _trend_ = 519.281, *p* _for trend_ < 0.001).

### 3.2. Demographic Characteristics of the Study Population

As shown in Table 3, 5890 residents with complete dietary intake data were enrolled in this study. Among them, 2106 were from coastal areas, 2031 from subcoastal areas, and 1753 from inland areas. Of these participants, 2734 were male, and 3156 were female. The age distribution included 868 individuals under 18 years old, 1713 aged between 18 and 44 years, 1636 aged between 45 and 59 years, and 1673 over 60 years old. Regarding urban-rural distribution, 3128 participants resided in urban areas, while 2762 were from rural areas. Among all participants, 3638 individuals (61.8%) reported consuming iodized salt, while 2252 (38.2%) reported not consuming iodized salt.

### 3.3. Dietary Iodine Intake among Residents in Zhejiang Province

As shown in Table 4, the median (P_25_, P_75_) dietary iodine intake among residents in Zhejiang Province was 142.05 (58.94, 237.11) μg/d. The dietary intakes in coastal, subcoastal, and inland areas were 75.66 (36.94, 172.02) μg/d, 153.42 (81.35, 251.47) μg/d, and 185.61 (118.13, 282.17) μg/day, respectively, with significant differences among the regions (χ^2^ = 588.592, *p* < 0.001).

Regarding regional differences, the median (P_25_, P_75_) dietary iodine intakes in urban and rural areas were 136.00 (55.14, 244.50) μg/d and 145.89 (63.58, 230.63) μg/d, respectively. No significant difference was found between the areas (*Z* = −1.249, *p* = 0.211).

Regarding sex differences, the median (P_25_, P_75_) dietary iodine intakes in males and females were 149.99 (62.13, 250.26) μg/d and 136.05 (56.03, 227.92) μg/d, respectively. Males had a significantly higher dietary iodine intake than females (*Z* = −3.957, *p* < 0.001).

Regarding differences based on the consumption of iodized salt, the median (P_25_, P_75_) dietary iodine intake was 191.98 (133.47, 283.93) μg/d in individuals consuming iodized salt, while it was 42.21 (26.33, 67.77) μg/d in those not consuming iodized salt. A significant difference in dietary iodine intake was observed between consumers and non-consumers (*Z* = −52.246, *p* < 0.001).

In the entire surveyed population, the proportions of individuals with dietary iodine levels falling below the EAR, between the EAR and RNI, between the RNI and UL, and exceeding the UL were 34.80%, 10.00%, 52.30%, and 2.90%, respectively, with significant difference among the regions (*χ*^2^ = 723.632, *p* < 0.001). The proportions of residents with a dietary iodine intake below the EAR by region, from the largest to the smallest, were as follows: coastal (54.80%), subcoastal (30.20%), and inland (16.00%) areas (*χ*^2^ _trend_ = 647.777, *p*_for trend_ < 0.001). The population of residents adhering to the RNI–UL range by region, from the largest to the smallest, were as follows: inland (69.40%), subcoastal (56.50%), and coastal (34.10%) areas (*χ*^2^_trend_ = 485.333, *p*_for trend_ < 0.001). The proportions of residents exceeding the safe intake range by region were as follows: inland (5.10%), coastal (2.30%), and subcoastal (1.60%) areas (*χ*^2^ _trend_ = 25.229, *p*_for trend_ < 0.001).

### 3.4. Contribution of Food Sources, Salt, and Drinking Water to Dietary Iodine Intake

**Figure 1:**
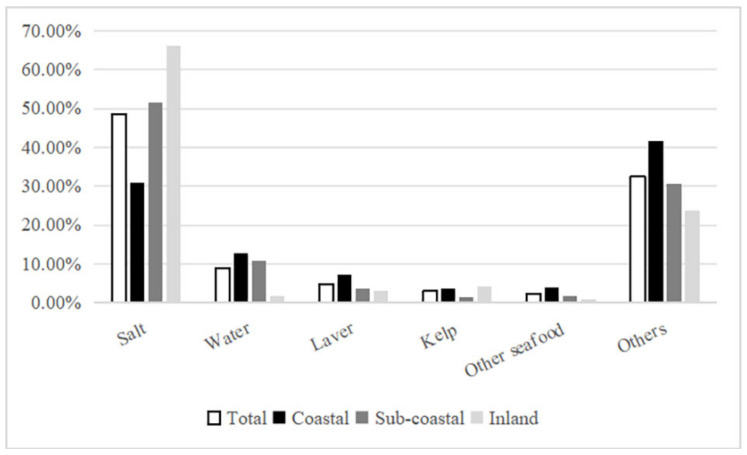
Proportion of iodine intake from various sources.

As shown in Figure 1, without considering cooking losses, the contributions of various food sources to dietary iodine intake among residents in Zhejiang Province, from the highest to the lowest, were as follows: salt (48.54%), other foods (32.46%), drinking water (8.84%), laver (4.82%), kelp (3.02%), and other seafood (2.32%).

As shown in Table 5, salt added during meal preparation stood out as the primary iodine source, comprising 48.54% of dietary intake. Notably, there was a significant regional variation in salt contribution as follows: inland areas (66.11%), subcoastal areas (51.61%), and coastal areas (30.95%) (*χ*^2^ = 853.271, *p* < 0.001). However, no significant sex-based difference in salt contribution was observed (*Z* = −0.679, *p* > 0.05). Furthermore, there was a significant difference in salt contribution between urban (46.23%) and rural (51.16%) areas (*Z* = −5.032, *p* < 0.001).

For individuals consuming iodized salt, the main contributors to dietary iodine, ranked from the highest to the lowest, were as follows: salt (73.34%), other foods (17.30%), drinking water (3.63%), laver (2.72%), kelp (2.10%), and other seafood (0.91%). Conversely, for individuals not consuming iodized salt, the main sources of dietary iodine were ranked in the following order: other foods (61.40%), drinking water (18.79%), laver (8.84%), other seafood (4.99%), and kelp (4.78%).

## 4. Discussion

The survey revealed that the median (P_25_, P_75_) dietary iodine intake among residents in Zhejiang Province was 142.05 (58.94, 237.11) μg/d, which is generally considered adequate. This value aligns closely with data reported in Fujian Province [20] and Xinjiang [21], China, as well as in Denmark [22], albeit it was significantly lower than the intake of 272.36 μg/d in 2010 in Zhejiang Province [23].

Furthermore, the analysis revealed a descending trend in the dietary iodine intake across different regions of Zhejiang Province, with inland areas having higher levels, followed by subcoastal areas and coastal areas. While residents in subcoastal and inland regions maintained sufficient and safe iodine intake levels, attention should be directed towards addressing the inadequate iodine intake among coastal area residents.

The survey also indicated significant sex-based differences in dietary iodine intake, with female residents consuming lower levels of iodine than male residents. Similar trends were observed in recent studies conducted in Denmark and Norway [22,24,25], possibly due to substantial variations in dietary habits and intake between the sexes. The higher food intake among males could be a contributing factor to their elevated dietary iodine levels.

Regarding water iodine levels, the median iodine concentration at each surveyed site in this study was below 40 μg/L. According to the “Definition and Demarcation of Iodine Deficient Areas and Iodine Adequate Areas” (WS/T669-2020) standard [19], all counties (districts, cities) in Zhejiang Province were categorized as environmental iodine-deficient areas. Most areas had low iodine content in their drinking water, consistent with the 2015 external environment water iodine monitoring results [26]. This suggests that the daily drinking water of residents in Zhejiang Province fails to meet the human body’s iodine requirements, necessitating additional iodine supplementation.

The median iodine concentration in salt across Zhejiang Province was 22.08 mg/kg, reflecting a decline of 6.72 mg/kg compared with the levels recorded in 2010 [27]. Inland areas and subcoastal regions had median household salt iodine concentrations of 23.42 mg/kg and 22.27 mg/kg, respectively, meeting the national standard range of 18–33 mg/kg. However, the median iodine concentration in salt in coastal regions was only 0.42 mg/kg, which is significantly lower than the national standard. The following were the consumption rates of qualified iodized salt by region, from the highest to the lowest: inland (84.2%), subcoastal (67.8%), and coastal (37.00%) areas; all values fell below the standards established by the National Criteria for Elimination of IDDs. Additionally, notable regional variations were observed. The iodine intake and rate of consumption of qualified iodized salt among residents in different regions were positively correlated, and the regions were ranked as follows based on both these factors: inland, subcoastal, and coastal areas. This corresponds to the differences observed in the consumption rates of qualified iodized salt across different regions in the 2010 iodine nutrition survey in Zhejiang Province [28], suggesting that the decline in residents’ dietary iodine intake may be associated with the reduction in salt iodine concentration and the decrease in the iodized salt consumption rate [20].

Residents in Zhejiang Province obtain iodine from various dietary sources, with the primary contributors ranked in the following order: salt (48.54%), other foods (32.46%), drinking water (8.84%), laver (4.82%), kelp (3.02%), and other seafood (2.32%). This suggests that salt remains the main source of dietary iodine for Zhejiang residents, although its contribution has decreased compared with the ratio of 74.92% reported in the 2010 iodine nutrition survey [23]. Furthermore, there are significant regional differences in salt contribution, which were ranked in the following order by region: inland (66.11%), subcoastal (51.61%), and coastal (30.95%) areas. These differences positively correlated with variations in the consumption of qualified iodized salt and iodine concentrations in salt across regions. Additionally, residents’ dietary iodine intake positively correlated with salt contribution, showing a similar pattern by region: inland areas > subcoastal areas > coastal areas. This further underscores the view that iodized salt remains the primary source of dietary iodine intake, aligning with the findings of recent studies in other regions of China [21,29].

Significant sex differences were observed in dietary iodine intake. The contribution rates of various foods did not differ between sexes. This observation may be attributed to family-based dietary habits in China, suggesting that sex differences in iodine intake are more related to the total amount of food consumed rather than specific food preferences. Additionally, the contribution rate of salt intake among urban residents was lower than that of rural residents. This phenomenon may be because rural areas still retain more traditional Chinese dietary habits, such as a preference for saltier pickled foods. Moreover, rural residents, who are often engaged in physical labor such as agricultural work, may believe that consuming more salt can replenish electrolytes lost through sweating and aid in maintaining physical strength, leading them to add more salt to their food.

While laver and kelp are known for their high iodine content, their consumption frequency and quantity among the surveyed individuals were relatively low [30], resulting in a limited contribution to dietary iodine. Despite other forms of seafood being consumed more frequently, their iodine content is not substantial, thus limiting their contribution to dietary iodine. Although residents in coastal areas consumed more seafood than those in subcoastal and inland areas, the dietary iodine intake in coastal regions was lower than that in subcoastal and inland areas, suggesting that seafood is not the primary source of dietary iodine. Furthermore, an analysis of the entire population, iodized salt consumers, and non-iodized salt consumers indicated that laver, kelp, and other seafood were not the main contributors to dietary iodine. This finding is consistent with that of research in Xinjiang [21], Tianjin [29], and other regions, confirming that laver, kelp, and other seafood are not the primary sources of dietary iodine.

Therefore, relying solely on regular dietary intake for iodine cannot fulfill the body’s daily iodine requirements. Dietary iodine intake is primarily influenced by the consumption of iodized salt, which in turn is affected by the qualified iodized salt consumption rate. In coastal regions, some residents choose not to use iodized salt and instead opt for non-iodized alternatives that are more readily available, thereby reducing their dietary iodine intake and the contribution of salt to iodine intake. Without the consumption of iodized salt, most residents will likely fail to meet the RNI for iodine, putting them at risk of iodine deficiency.

It is worth noting that 34.80% of the residents had a daily dietary iodine intake below the EAR, while only 2.90% had an iodine intake exceeding the UL. Specifically, in coastal areas, the dietary iodine intake of 54.80% fell below the EAR, attributed partly to less enthusiasm for iodized salt and reduced consumption of qualified iodized salt, leading to decreased iodine intake from salt. Furthermore, across coastal, subcoastal, and inland areas, the proportion of residents with dietary iodine intake exceeding the UL was generally small, suggesting that the health risks associated with iodine deficiency in Zhejiang Province far outweigh those of excessive iodine intake. Hence, without iodized salt consumption, the iodine obtained from current dietary and water sources in coastal regions fails to meet the recommended intake levels, highlighting the inadequacy of relying solely on iodine intake from water and food to satisfy the body’s demand. 

This study had two limitations. First, the iodine content in foods was estimated based on the “Chinese Food Composition” data. However, due to the absence of data for certain processed food categories and potential variations in food composition across different brands, the iodine content of certain food items had to be estimated based on that of similar items, which may not accurately reflect the iodine intake derived from the diet. Second, a substantial number of residents were recruited for participation in this study, and their total iodine intake was estimated based on their dietary intake over 3 consecutive days. However, we only recorded the amount of food consumed, without considering the actual food intake, as meals prepared from food items may not have been entirely consumed. Moreover, the employed method did not accurately capture iodine loss during the cooking process. Consequently, the actual dietary iodine intake may be lower than the values derived from the survey, potentially resulting in an overestimation of iodine intake.

The human body has limited storage capacity for iodine, so iodine supplementation should follow the principles of long-term, daily, and lifelong. Based on the results of this survey, we propose several suggestions. First, iodized salt is still the most important source of dietary iodine. Government departments should strengthen the production and sale management of non-iodized salt to make it less readily available than iodized salt. Second, health education should be strengthened to inform residents, especially those in coastal areas, of the current status of iodine nutritional sources in Zhejiang Province (it is generally believed that seafood is not the main source of dietary iodine) and raise residents’ awareness of the importance of eating iodized salt. In addition, pregnant women and lactating mothers should increase their consumption of iodine-rich seafood such as kelp and laver, and supplement iodine-containing preparations according to medical advice.

## 5. Conclusions

Zhejiang Province is classified as an area with environmental iodine deficiency, indicating a persistent natural iodine deficiency. While overall dietary iodine intake is adequate, it is notably insufficient and declining in coastal regions. Iodized salt remains the primary source of dietary iodine for residents, with the contributions of laver, kelp, and other seafood being limited. Therefore, Zhejiang Province must continue to prioritize comprehensive prevention and control measures for IDDs, centered on iodized salt. Additionally, through various approaches such as health education, efforts should be made to reverse the declining trend in the consumption of iodized salt, thereby ensuring the health of residents and preventing the resurgence of IDDs.

## Figures and Tables

**Table 1:** Reference dietary iodine intake standards for Chinese residents.

Variable	EAR	RNI	UL
0~	-	85 *	-
0.5~	-	115 *	-
1~	65	90	-
4~	65	90	200
7~	65	90	300
11~	75	110	400
14~	85	120	500
18~	85	120	600
Pregnancy	160	230	600
Lactation	170	240	600

* Adequate Intake (Al); references for the estimated average requirement (EAR), recommended nutrient intake (RNI), and tolerable upper intake level (UL) for iodine were obtained from the “Dietary Reference Intakes for Chinese Residents”.

**Table 2:** Water iodine, salt iodine situation.

Geographic Region	Water Iodine		Salt Iodine					
	Total Sample	Iodine Content M (P_25_, P_75_), (μg/L)	Total Sample	Iodine Content M (P_25_, P_75_), (mg/kg)	Coverage Rate (%)	Qualified Rate (%) ^4^		
						Non-Iodized	Adequately Iodized	Inadequately or Excessively Iodized
Coastal	63	3.2 (2.5, 5.4) ^a^	970	0.42 (0.00, 22.70) ^a^	41.60	58.40	37.00	4.60
Sub-coastal1	84	3.8 (1.2, 4.9) ^b^	918	22.27 (0.00, 24.61) ^b^	71.60	28.40	67.80	3.80
Inland	104	1.0 (0.4, 2.0) ^c^	1159	23.42 (21.04, 25.12) ^c^	88.10	11.90	84.20	3.90
H ^1^		98.942		371.885				
*χ^2^* _trend_ ^2^						519.281	504.765	0.721
*p*-Value ^3^		<0.001		<0.001		<0.001	<0.001	0.396
Total	251	2.2 (0.9, 4.0)	3047	22.08 (0.00, 24.51)	68.30	31.70	64.20	4.10

M: 50th percentile (median); P_25_: 25th percentile, the first quartile; P_75_: 75th percentile, the third quartile; ^1^ H-value of the Kruskal–Wallis *H* test; ^2^ chi-square trend value of the chi-square trend test; ^3^ *p*-value of the Kruskal–Wallis H test and chi-square trend test; ^4^ according to the standard of “Iodine Content of Edible Salt” (GB 26878-2011), the salt samples were determined to be non-iodized salt with an iodized salt content of <5 mg/kg, unqualified iodized salt with an iodized salt content of <18 mg/kg or >33 mg/kg, and qualified iodized salt with 18–33 mg/kg; ^a,b,c^: subgroups with different letters indicate significant differences (*p* < 0.05).

**Table 3:** Demographic characteristics of the 5890 residents.

Variable	Total Sample, n (%)
Geographic region	
Coastal	2106 (35.80)
Sub-coastal	2031 (34.50)
Inland	1753 (29.80)
Distribution	
Urban	3128 (53.10)
Rural	2762 (46.90)
Gender	
Male	2734 (46.40)
Female	3156 (53.60)
Age (years)	
<18	868 (14.70)
18–44	1713 (29.10)
45–59	1636 (27.80)
≥60	1673 (28.40)
Salt iodine	
Iodized	3865 (65.60)
Non-iodized	2025 (34.40)

**Table 4:** Dietary iodine intake among residents in Zhejiang Province and its relationship with reference intake levels.

Variable	Total Sample	M (P_25_, P_75_), (μg/d)	<EAR	EAR–RNI	RNI–UL	>UL
Total	5890	142.05 (58.94, 237.11)	2050 (34.80)	587 (10.00)	3082 (52.30)	171 (2.90)
Geographic region						
Coastal	2106	75.66 (36.94, 172.02) ^a^	1155 (54.80) ^a^	184 (8.70) ^a^	719 (34.10) ^a^	48 (2.30)
Sub-coastal	2031	153.42 (81.35, 251.47) ^b^	614 (30.20) ^b^	237 (11.70) ^b^	1147 (56.60) ^b^	33 (1.60)
Inland	1753	185.61 (118.13, 282.17) ^c^	281 (16.00) ^c^	166 (9.50) ^a^	1216 (69.40) ^c^	90 (5.10)
Distribution						
Urban	3128	136.00 (55.14, 244.50)	1146 (36.60)	301 (9.60)	1597 (51.10)	84 (2.70)
Rural	2762	145.89 (63.58, 230.63)	904 (32.70)	286 (10.40)	1485 (53.80)	87 (3.10)
Gender						
Male	2734	149.99 (62.13, 250.26) ^a^	923 (33.80)	249 (9.10)	1474 (53.90)	88 (3.20)
Female	3156	136.05 (56.03, 227.92) ^b^	1127 (35.70)	338 (10.70)	1608 (51.00)	83 (2.60)
Salt iodine						
Iodized	3865	191.98 (133.47, 283.93) ^a^	386 (10.00) ^a^	449 (11.60) ^a^	2887 (74.70) ^a^	143 (3.70) ^a^
Non-iodized	2025	42.21 (26.33, 67.77) ^b^	1664 (82.20) ^b^	138 (6.80) ^b^	195 (9.60) ^b^	28 (1.40) ^b^

M: 50th percentile (median); P_25_: 25th percentile, the first quartile; P_75_: 75th percentile, the third quartile; references for the estimated average requirement (EAR), recommended nutrient intake (RNI), and tolerable upper intake level (UL) for iodine were obtained from the “Dietary Reference Intakes for Chinese Residents”; ^a,b,c^ subgroups with different letters indicate significant differences (*p* < 0.05).

**Table 5:** Contribution of different dietary sources to dietary iodine intake (%).

Variable	Salt	Drinking Water	Laver ^3^	Kelp ^4^	Other Seafood ^5^	Other Foods ^6^
Total	48.54	8.84	4.82	3.02	2.31	32.46
Geographic region						
Coastal	30.95 ^a^	12.73 ^a^	7.14 ^a^	3.61 ^a^	4.03 ^a^	41.55 ^a^
Sub-coastal	51.61 ^b^	10.89 ^a^	3.80 ^b^	1.34 ^b^	1.75 ^b^	30.61 ^b^
Inland	66.11 ^c^	1.80 ^b^	3.23 ^b^	4.27 ^c^	0.90 ^c^	23.69 ^c^
*p*-Value ^1^	<0.001	<0.001	<0.001	<0.001	<0.001	<0.001
Distribution						
Urban	46.23	9.22	4.93	2.81	3.18	33.62
Rural	51.16	8.41	4.71	3.26	1.33	31.14
*p*-Value ^2^	<0.001	<0.001	0.462	0.733	<0.001	<0.001
Gender						
Male	48.20	8.91	5.01	2.92	2.33	32.63
Female	48.84	8.78	4.66	3.11	2.30	32.31
*p*-Value ^2^	0.497	0.112	0.097	0.379	0.416	0.709
Salt						
Iodized	73.34	3.63	2.72	2.10	0.91	17.30
Non-iodized	1.20	18.79	8.84	4.78	4.99	61.40
*p*-Value ^2^	<0.001	<0.001	<0.001	<0.001	<0.001	<0.001

^1^*p*-value of the Kruskal–Wallis *H* test; ^2^ *p*-value of the Mann–Whitney *U* test; ^3^ laver, including laver and its products; ^4^ kelp, including kelp (fresh), kelp (dried), kelp (soaked), sea cabbage (fresh, Gu Xiang brand), and so on; ^5^ other seafood, including sea fish, shrimp, crabs, and shellfish; ^6^ other foods including cereals, tubers, dried beans, vegetables, fruits, meat, milk, eggs, and freshwater fish; ^a,b,c^ subgroups with different letters indicate significant differences (*p* < 0.05).

## Data Availability

The data presented in this study are available on request from the corresponding author. The data are not publicly available due to confidentiality concerns.
